# Diagnostic accuracy of fine needle aspiration biopsy versus postoperative histopathology for diagnosing thyroid malignancy

**DOI:** 10.1002/edm2.373

**Published:** 2022-09-23

**Authors:** Aziz Aliyev, Irada Aliyeva, Francesco Giammarile, Narmin Talibova, Gunay Aliyeva, Fuad Novruzov

**Affiliations:** ^1^ Department of Head and Neck Surgery, Azerbaijan National Centre of Oncology Baku Azerbaijan; ^2^ Department of Internal Medicine, Azerbaijan Medical University Baku Azerbaijan; ^3^ International Atomic Energy Agency Vienna Austria; ^4^ Service de Médecine Nucléaire Lumen, Centre Léon Bérard Lyon France; ^5^ Department of Medical Oncology, Azerbaijan National Centre of Oncology Baku Azerbaijan; ^6^ Department of Nuclear Medicine, Azerbaijan National Centre of Oncology Baku Azerbaijan

**Keywords:** Bethesda classification, fine needle aspiration biopsy, thyroid nodules

## Abstract

**Introduction:**

Ultrasound‐guided fine needle aspiration biopsy (FNAB) is currently widely used for the initial screening of patients with thyroid nodules enabling prevention of unnecessary surgery. The purpose of this study was to retrospectively analyse the diagnostic accuracy of thyroid FNAB compared with postoperative histopathology of a large cohort from Azerbaijan.

**Methods:**

We evaluated the FNAB results of 738 patients who underwent thyroid surgery at the National Centre of Oncology in Azerbaijan. The measures of diagnostic accuracy were calculated for the ultrasound‐guided preoperative FNAB results (based on the six diagnostic categories of the Bethesda classification) compared with postoperative histopathologic results (benign or malignant) for correspondent areas.

**Results:**

Considering both DC V and DC VI categories (387 cases) as ‘cytologic‐positive’ and DC II category (72 cases) as ‘cytologic‐negative’, we found 14 false‐positive and 10 false‐negative results. The sensitivity, specificity, positive predictive value (PPV), negative predictive value (NPV) and diagnostic accuracy were 97.4%, 86.1%, 96.4%, 81.6% and 94.8%, respectively. Conversely, when considering only the DC VI category as ‘cytologic‐positive’, the sensitivity, specificity, PPV, NPV and diagnostic accuracy of FNA were 93.2%, 100%, 100%, 81.6% and 97.1%, respectively.

**Conclusions:**

The results of our cohort demonstrated high levels of diagnostic accuracy, supporting FNAB's role as a reliable diagnostic tool in the preoperative evaluation of thyroid nodules. The sensitivity, specificity, NPV, PPV and accuracy of thyroid FNAB in our institution were comparable with those of other institutions.

## Graphical abstract

Ultrasound‐guided fine needle aspiration biopsy (FNAB) is currently widely used for the initial screening of patients with thyroid nodules enabling prevention of unnecessary surgery. The purpose of this study was to retrospectively analyse diagnostic accuracy of thyroid FNAB compared to postoperative histopathology of a large cohort from Azerbaijan.

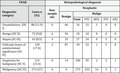

## 1 BACKGROUND

Thyroid nodules are common in the general population with the global incidence ranging between 40,000 and 71,000 per 100,000 persons.^1^, ^2^ The majority of thyroid nodules are benign and the risk for malignancy in asymptomatic nodules is 0.45%–13%.^1^, ^2^, ^3^ Fine‐needle aspiration (FNA) cytology (FNAC) is a reliable and cost‐effective method for thyroid nodule evaluation.^4^ FNA biopsy (FNAB) defines cases when surgery is required to be performed and decreases the overall incidence of thyroidectomy performed in benign cases. Studies have suggested a high sensitivity and specificity for thyroid malignancies prediction, ranging between 65%–98% and 72%–100%, respectively.^5^ Unfortunately, FNAB may have some false‐negative and false‐positive outcomes. Factors implicated for this rate may include specimen adequacy, sampling technique, the skill of the physician who performed an aspiration and experience of a pathologist.^6^

The purpose of this study was to evaluate the measures of diagnostic accuracy of FNAB performed at the National Center of Oncology in Azerbaijan by comparing it with postoperative histological diagnoses and define possible causes of errors leading to false‐negative and false‐positive results in order to better identify the factors that may affect the accuracy.

## 2 METHODS

The study covered the retrospective analysis of results obtained from January 2016 to December 2019. Data from 959 patients who had thyroid surgery in the National Centre of Oncology were reviewed from electronic medical records. We considered only patients with surgical indication of suspected thyroid malignancy who underwent FNAB in the same institution (*n* = 738). The patient's age ranged between 6 and 81 (median 46), and the sex ratio was 637/101 (female to male).

All performed FNABs were ultrasound‐guided and ultrasound risk stratification was performed according to TI‐RADS (thyroid imaging reporting and data system) and collected samples were smeared, stained with haematoxylin–eosin and submitted for cytological interpretation.

All FNABs were classified in accordance with the Bethesda system for reporting thyroid cytopathology, including six diagnostic categories (DCs): non‐diagnostic or unsatisfactory (DC I); benign (DC II); atypia of undetermined significance or follicular lesion of undetermined significance (DC III); suspicious for follicular neoplasm or follicular neoplasm (DC IV); suspicious for malignancy (DC V) and malignant (DC VI). Adequacy was determined based on the standard of Bethesda criteria.^7^

Preoperative ultrasound‐guided FNAB results were compared with postoperative histopathological results (benign or malignant) for correspondent areas to obtain the measures of diagnostic accuracy for FNAB.

The level of thyroglobulin was measured in all patients before surgery, and the results were interpreted according to the established gender‐specific reference ranges.^8^ Data were statistically analysed using IBM SPSS Statistics, in order to evaluate sensitivity, specificity, positive predictive value (PPV), negative predictive value (NPV) and diagnostic accuracy of the study.

## 3 RESULTS

According to the thyroid FNAB results, 88 cases (11.9%) were interpreted as unsatisfactory (DC I), 72 cases (9.8%) as benign (DC II), 63 cases (8.5%) as atypia (DC III), 128 cases (17.4%) as follicular lesions of undetermined significance (DC IV), 114 cases (15.4%) as suspicious for malignancy (DC V) and 273 cases (37.0%) as malignant samples (DC VI). Final histopathological diagnosis was concluded in 273 cases (37%) for benign and non‐neoplastic lesions and in 465 cases (63%) for malignant neoplasms. Detailed pathology characteristics are shown in Table 1.

**TABLE 1:** Pathological characteristics of the cases

FNAB		Histopathological diagnosis						
Diagnostic category	Cases, *n* (%)	Non‐neoplastic	Neoplastic					
			Benign	Malign				
				Total	PTC	MTC	FTC	ATC
Unsatisfactory (DC I)	88 (11.9)	6	66	16	15	1	0	0
Benign (DC II)	72 (9.8)	6	56	10	10	0	0	0
Atypia (DC III)	63 (8.5)	3	33	27	24	0	3	0
Follicular lesion of undetermined significance (DC IV)	128 (17.4)	2	87	39	33	1	5	0
Suspicious for malignancy (DC V)	114 (15.4)	0	14	100	95	2	2	1
Malignancy (DC VI)	273 (37)	0	0	273	253	14	5	1

*Note*: ATCX, anaplastic thyroid carcinoma; FTC, follicular thyroid carcinoma; MTC, medullary thyroid carcinoma; PTC, papillary thyroid carcinoma.

For a first analysis, the cases which were considered by FNAB as malignant (DC VI) or suspicious for malignancy (DC V) were defined as ‘cytologic positive’. From a total of 387 lesions, post‐surgical histology revealed indeed 14 benign lesions, judged as false‐positive. Similarly, from the 72 cases considered by FNAB as benign and defined as ‘cytologic‐negative’, after surgery 10 lesions were judged as false‐negative. In this case, the sensitivity, specificity, PPV, NPV and diagnostic accuracy were 97.4%, 86.1%, 96.4%, 81.6% and 94.8%, respectively.

A histopathologic review of the false‐negative cases revealed that 7 out of 10 cases were papillary thyroid microcarcinoma (PTMC). In three false‐negative cases (30%), malignancy was diagnosed in multinodular goitre. In seven cases, tumours with the size of 1 cm or smaller were classified as T1a, 1 case as T1b, 2 cases were T3 according to the TNM Classification of Malignant Tumours, 8th Edition.^9^ Only in 2 of 10 cases, tumour cells were detected also in lymph nodes, and they were classified as N1a. Postoperative histological examination revealed all tumours to be papillary cancer.

All false‐positive cases were considered by FNAB as malignancy suspicious (DC V). Postoperative histopathological diagnoses of these cases revealed Hurthle cell adenoma in 3 cases, multinodular goitre in 3 cases, microfollicular adenoma in 4 cases, adenomatous hyperplasia in 2 cases and macrofollicular adenoma in 2 cases. Out of 14, 9 cases were false‐positive cases were thyroiditis; while 4 cases were pure thyroiditis, 5 cases of Hashimoto thyroiditis and 1 case of granulomatous thyroiditis. Therefore, when defining only the malign category (DC VI) as ‘cytologic‐positive’, the sensitivity, specificity, PPV, NPV and diagnostic accuracy of FNAB were 93.2%, 100.0%, 100%, 81.6% and 97.1%, respectively.

Pre‐ and post‐operative thyroglobulin levels were measured due to its known high sensitivity for thyroid malignancy^8^; however, one‐way ANOVA revealed no statistically significant difference in thyroglobulin levels of different Bethesda categories (*f*‐ratio = 0.66094; *p* = .6194) and decrease in postoperative thyroglobulin levels was also not significant (*p* = .0899). The level of thyroglobulin was increased in 3 (30%) patients while it was normal in 7 (70%) patients in false‐negative cases. In false‐positive cases, on the contrary, thyroglobulin was increased in 4 (28.6%) patients and was normal in 10 (71.4%).

## 4 DISCUSSION

Thyroid nodules can be caused by different benign and malign disorders.^1^ Initial assessment of the patients with a thyroid nodule includes a detailed history and physical examination. Thyroid ultrasound is performed to confirm the presence of nodules, to evaluate additional nodes and cervical lymph nodes and to assess for sonographic features. The next step is a FNA biopsy, which is considered a gold standard for thyroid nodule evaluation.^4^, ^5^ FNAB is an economical, minimally invasive and accurate method. It may be performed by palpation or with ultrasound guidance. Needle biopsy of simple cysts is not recommended. In this study, all FNABs were ultrasound guided. FNAB is recommended for the thyroid nodules larger than 1 cm; it is not usually performed on nodules smaller than that. The interpretation of the FNAB smears may be influenced by different factors such as technique and specimen preparation, the skill of the operator and the cytopathologist's expertise.^6^ Our multidisciplinary team consists of two surgeons, one pathologist who specializes in thyroid disorders, one nuclear medicine specialist, two endocrinologists and one radiologist.

According to the literature, category V (malignant suspicious) and category VI (malignant) show around 45%–60% and 94%–96% risk of malignancy, respectively, whereas our results showed 87.7% malignancy confirmation through postoperative histopathology in DC V and 100% in DC VI.^5^ Both categories need surgical excision according to the American Thyroid Association guidelines. For that reason, in our study, we considered only categories V and VI as ‘cytologic positive’. When both these categories were considered as positive findings the sensitivity, specificity, PPV, NPV and diagnostic accuracy were 97.4%, 86.1%, 96.4%, 81.6% and 94.8%, respectively. Interestingly, all the false‐positive cases were malignancy suspicious (DC V) according to the FNAC results. Thus, our results clearly indicate that the category suspicious for malignancy (DC V) cannot be considered highly reliable for malignancy diagnosis. If this category is not included and only the malignant category (DC VI) was considered ‘cytologic‐positive’, sensitivity, specificity, PPV, NPV and diagnostic accuracy of FNAC were 93.2%, 100.0%, 100%, 81.6% and 97.1%, respectively.

The ultrasound results of all the cases which were suspicious for malignancy (DC V) by FNAC were clinically re‐evaluated. All high‐risk nodules with characteristic ultrasonographic features (low echo density, microcalcifications, irregular borders and intense vascularization) were re‐examined by the pathologist.

Our findings closely correspond to the results of a systematic review recently published by Zhu et al.,^10^ reporting 93.5% (our 94.8%) and 97.5% (our 97.1%) diagnostic accuracy in DC V + DC VI and DC VI only groups, respectively.

The low false‐negative rate (FNR) of FNAC makes it a reliable test for guiding the operative decision‐making process. According to most studies, FNRs of FNAC is <5%.^11^ Our negative thyroid FNAC results were reliable, as the FNR was 2.6%. The analyses of the tumour size of the patients with false‐negative results revealed PTMC in seven patients. This result was close to the previous studies which suggest that some pathologists consider nodules smaller than 1 cm as false‐negative.^12^ According to the previous reports, FNRs range between 17% and 19.3% for thyroid nodules of 3–4 cm.^13^, ^14^ In our study, only two cases with false‐negative results were larger than 2 cm (two cases were T3 according to the TNM Classification of Malignant Tumours, 8th Edition).

The study showed that hyperplastic and adenomatous nodules were the most important factors in the false‐positive diagnosis, as they can be misdiagnosed as suspicious for papillary thyroid carcinoma. It means that benign thyroid nodules with papillary hyperplasia can be a challenge not only in cytology diagnosis, but also in surgical pathology by mimicking classical papillary thyroid carcinoma. The second factor for misdiagnosis was Hurthle cell adenoma. Previous study suggests that papillary thyroid carcinoma cases with Hurthle cell‐like cells may cause the diagnostic errors. Nuclear budding and granular chromatin of Hurthle cells are important for the prediction of the outcome of neoplasm and malignancy.^15^

Considering its high diagnostic accuracy which correlates with previous reports, we recommend that treatment stratification of ultrasonographically suspect nodules should be performed by FNAB before any surgery in the thyroid gland.^16^

## 5 CONCLUSIONS

The sensitivity, specificity, NPV, PPV and accuracy of thyroid FNAB in our institution were comparable with those of other institutions. The reason for false‐positive diagnoses in our study was related to thyroiditis cases which include pure thyroiditis, Hashimoto thyroiditis and granulomatous thyroiditis. The awareness of pathologists regarding these pitfalls can minimize false‐negative and false‐positive diagnoses. FNAB is considered a screening procedure; thus, particular attention should be given to minimizing false‐negative diagnoses.

## AUTHOR CONTRIBUTIONS

**Aziz Aliyev:** Conceptualization (lead); data curation (lead); methodology (lead); supervision (lead); writing – original draft (lead). **Irada Aliyeva:** Methodology (supporting); supervision (supporting). **Francesco Giammarile:** Writing – review and editing (supporting). **Narmin Talibova:** Data curation (equal); formal analysis (equal); methodology (equal); validation (equal); writing – original draft (equal). **Gunay Aliyeva:** Writing – original draft (equal); writing – review and editing (supporting). **Fuad Novruzov:** Supervision (equal); visualization (supporting); writing – review and editing (supporting).

## CONFLICT OF INTEREST

None.

## ETHICS STATEMENT AND CONSENT TO PARTICIPATE

The trial was approved by the Ethics Committee of Azerbaijan National Centre of Oncology (EKQ/002.21). The study was performed fulfilling the principles of Helsinki. Declaration and all subjects who agreed to participate in the study have signed informed consent.

## Data Availability

Data are available upon request. Please contact the corresponding author.
